# Congenital Absence of Caecum and Ascending Colon

**Published:** 1913-01

**Authors:** 


Congenital Absence of Caecum and Ascending Colon. P. L. Mummery, *Annals of Surg.*, page 844, 1912. The lower four inches of the ileum was dilated and joined the right portion of the transverse colon directly, without an ileo-caecal valve but with a rudimentary appendix. No inconvenience due to the anomaly. 5 similar cases in literature.

